# Supplementary material for: Schistosoma mansoni treatment reduces HIV entry into cervical CD4+ T cells and induces IFN-I pathways
Source: Nat Commun. 2019 May 24;10:2296. doi: 10.1038/s41467-019-09900-9 (PMC6534541; doi:10.1038/s41467-019-09900-9)
Supplement: Supplementary file 1 — Supplementary Information [file 41467_2019_9900_MOESM1_ESM.pdf]

***Schistosoma mansoni* treatment reduces HIV entry into cervical CD4+ T cells and induces IFN-I pathways.**

Sergey Yegorov[1]*, Vineet R. Joag[1], Ronald M. Galiwango[1], Sara V. Good[2,3], Juliet Mpendo[4], Egbert Tannich[5], Andrea K. Boggild [6,7], Noah Kiwanuka[4,8], Bernard S. Bagaya[4,9], Rupert Kaul[1,6]

Supplementary Note 1. Demographic data

Supplementary Table 1. Lakeshore communities that participated in the study.

Village/Community	Distance from Lake (km)	N of recruited participants	% of recruited participants
Abaita	1.6	1	2.9
Banga	0.5	8	23.5
Kitoro	2.8	3	8.8
Kiwafu	1.7	2	5.9
Lugonjo	1.0	9	26.5
Lunyo	1.9	5	14.7
Nakiwogo	0.5	5	14.7
Nkumba	2.2	1	2.9

Supplementary Note 2. Schistosomiasis diagnostic data

Supplementary Table 2. The scoring system used to semi-quantitatively assess schistosomiasis burden based on the relative brightness of the urine POC-CCA test band.

Urine CCA test score	Brightness of "test" band relative to "control" band
**0.5**	< (very faint band)
**+1**	< (less bright, but clear)
**+2**	= (equally bright)
**+3**	> (brighter)

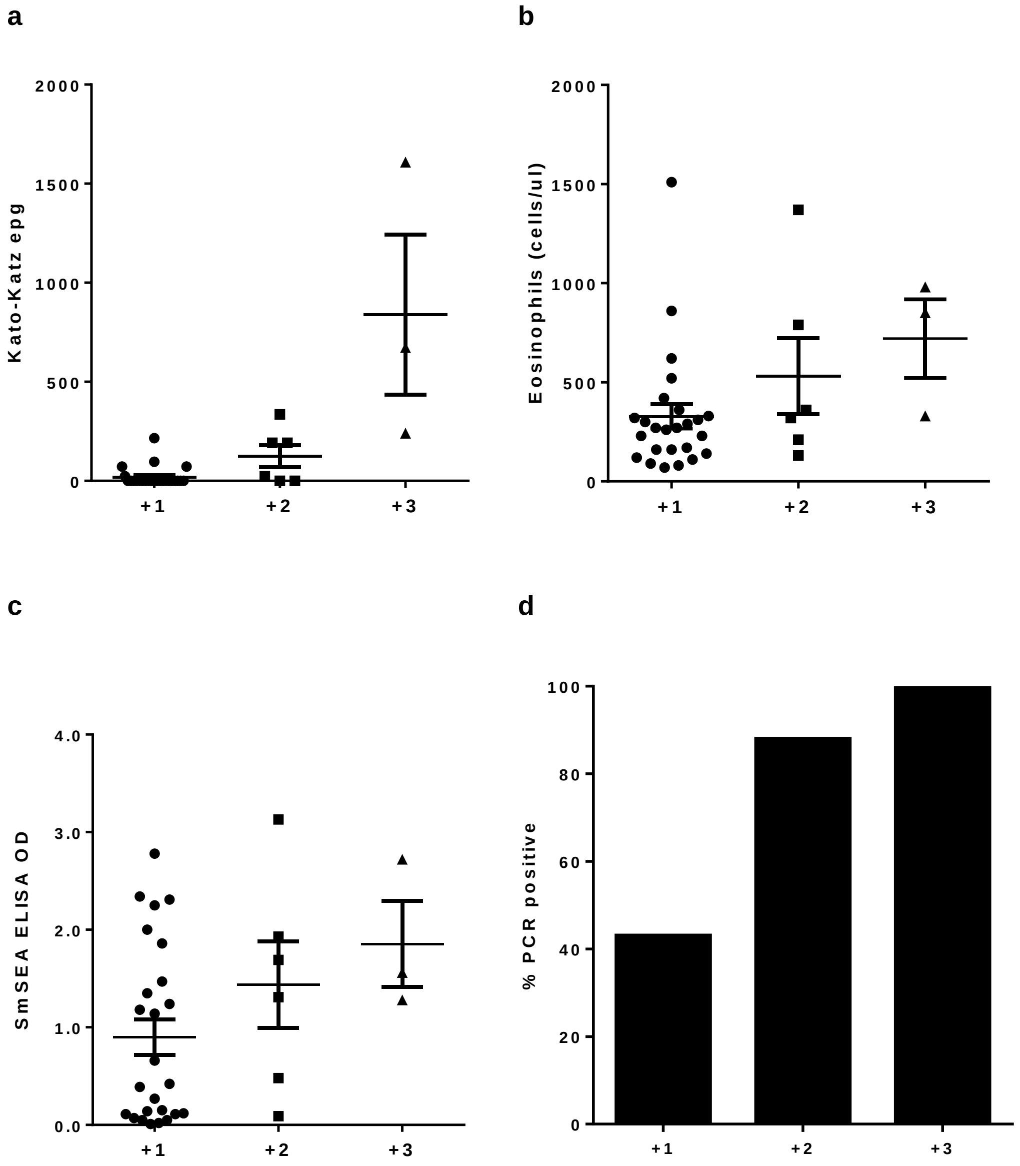

Supplementary Fig. 1. Relationship between the urine CCA scores and the a) Kato Katz microscopy, b) circulating eosinophil counts, c) *Sm*SEA ELISA and d) PCR positivity results. Epg: *Sm* eggs per gram of stool. OD: ELISA optic density units. In a, b and c, bars represent means and SEM. Source data are provided as a Source Data file.

Supplementary Table 3 Diagnostic outcomes of *S. mansoni*-specific PCR and serology in all CCA+ participants (n=34). Source data are provided as a Source Data file.

	***Sm* PCR/serology + group**			***Sm* PCR and serology - group**
	**PCR+, serology+**	**PCR+, serology-**	**PCR-, serology+**	
**N of participants**	20	1	3	10

Supplementary Table 4. Summary of CCA score changes associated with schistosomiasis treatment in the trial.

CCA score change	V2 minus V1		V3 minus V1	
	Participant N	%	Participant N	%
**-3**	NA	NA	1	4.17
**-2.5**	3	10.35	2	8.34
**-2**	1	3.45		0
**-1.5**	2	6.9	3	12.5
**-1**	5	17.25	5	20.84
**-0.5**	15	51.73	11	45.84
**0**	2	6.9	2	8.34
**1**	1	3.45	NA	NA
**Total**	29	100	24	100

Supplementary Table 5. Median changes in CCA scores associated with treatment of schistosomiasis in the entire CCA+ cohort and in the *Sm* PCR/serology+ subset of participants.

**Study visit**	**Parameter**	**All CCA+ participants (baseline n=34)**	***Sm* PCR/serology+ participants (baseline n=24)**
One month post-treatment (V2)	**CCA score, median change (V2-baseline)**	-0.5* (p<0.001)^	-0.5* (p<0.001)
Two months post-treatment (V3)	**CCA score, median change (V3-baseline)**	-0.5* (p<0.001)^	-1.0* (p<0.001)

*Significant change compared to baseline visit, at p≤0.05, as assessed by Wilcoxon signed rank test.
^ Out of 29 participants at V2, CCA scores were reduced compared to baseline in all but 3 participants (did not change in 2 and increased in 1); out of 24 participants at V3, CCA scores were reduced compared to baseline in all but 2 participants (did not change in 2).

Supplementary Note 3. Full blood count and SmSEA antibody titre data

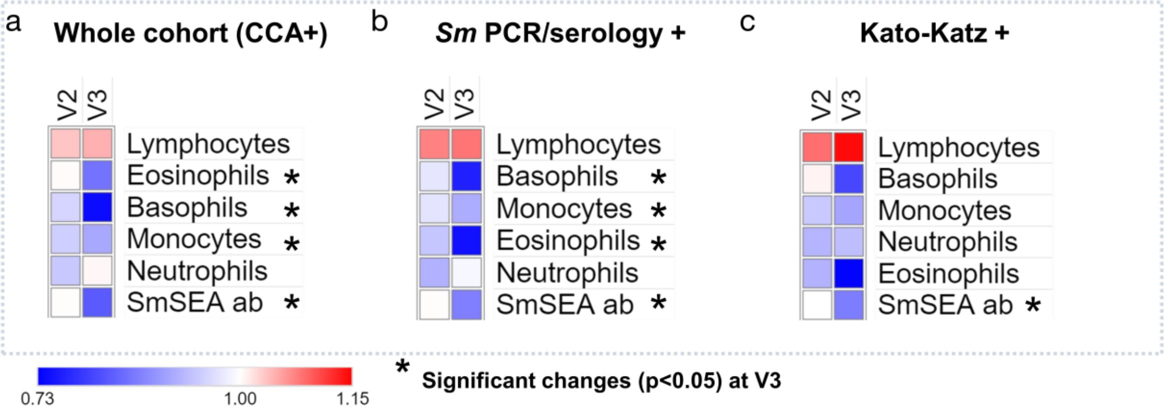

Supplementary Fig. 2. Changes associated with schistosomiasis treatment of circulating immune cell subset numbers derived from full blood counts and anti-*Sm* soluble egg antigen antibody titres (SmSEA ab) in a) the entire CCA+ cohort (n=34), b) *Sm* PCR/serology+ participants (n=24) and c) Kato-Katz+ participants (n=12). Fold changes were calculated relative to baseline visit (V1) and represented as geometric means of V2/V1 and V3/V1 ratios for each parameter. Cell subset changes were sorted in descending order (highest fold change at V2 first). *Significant changes compared to baseline visit were assessed by Wilcoxon signed rank test p≤0.05. The files for interactive viewing using Morpheus are available for download at: https://figshare.com/s/9e5fd3fcc4e5ac0d43d1. Source data are provided as a Source Data file.

Supplementary Table 6. Sub-grouping of participants by the direction of *Sm*SEA ab titre change associated with schistosomiasis treatment in all participants CCA+ at baseline.

	**V2 minus V1**		**V3 minus V1**	
**Direction of optic density change**	**N**	**%**	**N**	**%**
**Negative**	10	34.5	20	83.3
**No Change**	2	6.9	2	8.3
**Positive**	17	58.6	2	8.3
**Total**	29		24	

Supplementary Note 4. Flow cytometry and HIV entry assay data

Supplementary Table 7. Description of the antibodies and live-dead dye used in flow cytometry assays.

Fluorochrome/Dye	Marker	Clone	Manufacturer	Cat#
**BV605**	**CCR7 (CD197)**	**G043H7**	Biolegend	**353224**
**BV650**	**CD4**	**SK3**	BD	**563875**
**BV711**	**CD38**	**HIT2**	Biolegend	**303528**
**BV785**	**CD3**	**OKT3**	Biolegend	**317330**
**Far Red**	**Live dead**	**n/a**	Thermo Fisher/Invitrogen	**L10120**
**AF700**	**HLA-DR**	**L243**	Biolegend	**307626**
**PE CF594**	**CCR5 (CD195)**	**2D7**	BD	**562456**
**PE-Cy5**	**Integrin B7**	**FIB504**	BD	**551059**
**PE-Cy7**	**CD69**	**FN50**	Biolegend	**310912**

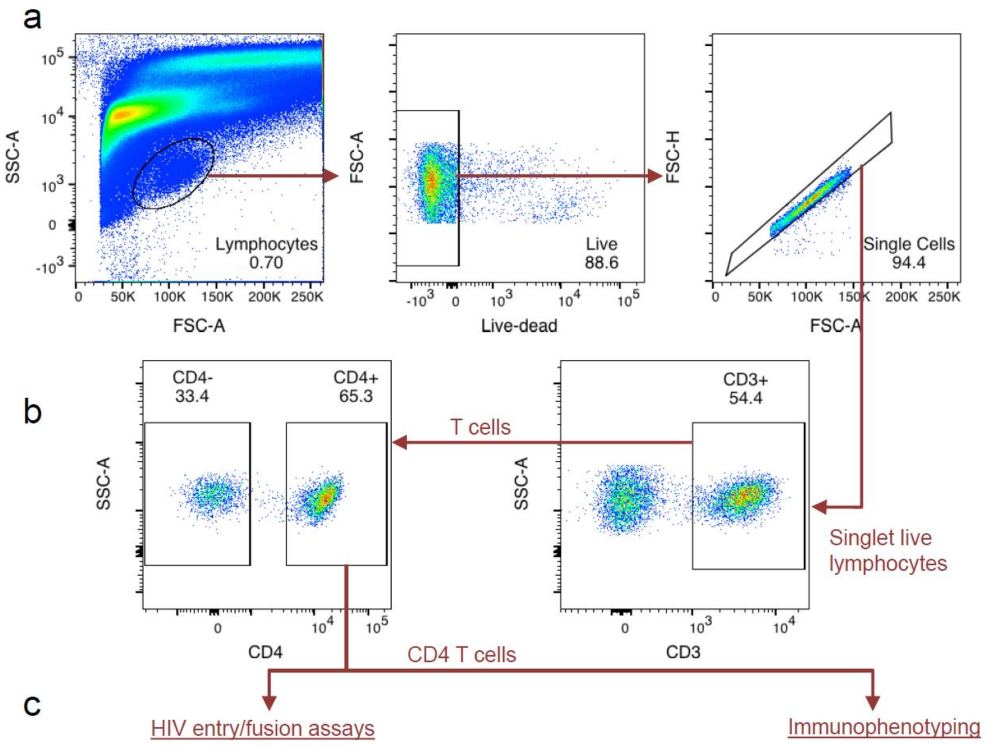

Supplementary Fig. 3. Representative flow cytometry plots and gating strategy for cervical mononuclear cells. a) Gating on single live lymphocytes; b) Gating on CD4 T cells; c) See Main text Fig. 2 for representative HIV entry and immunophenotyping plots.

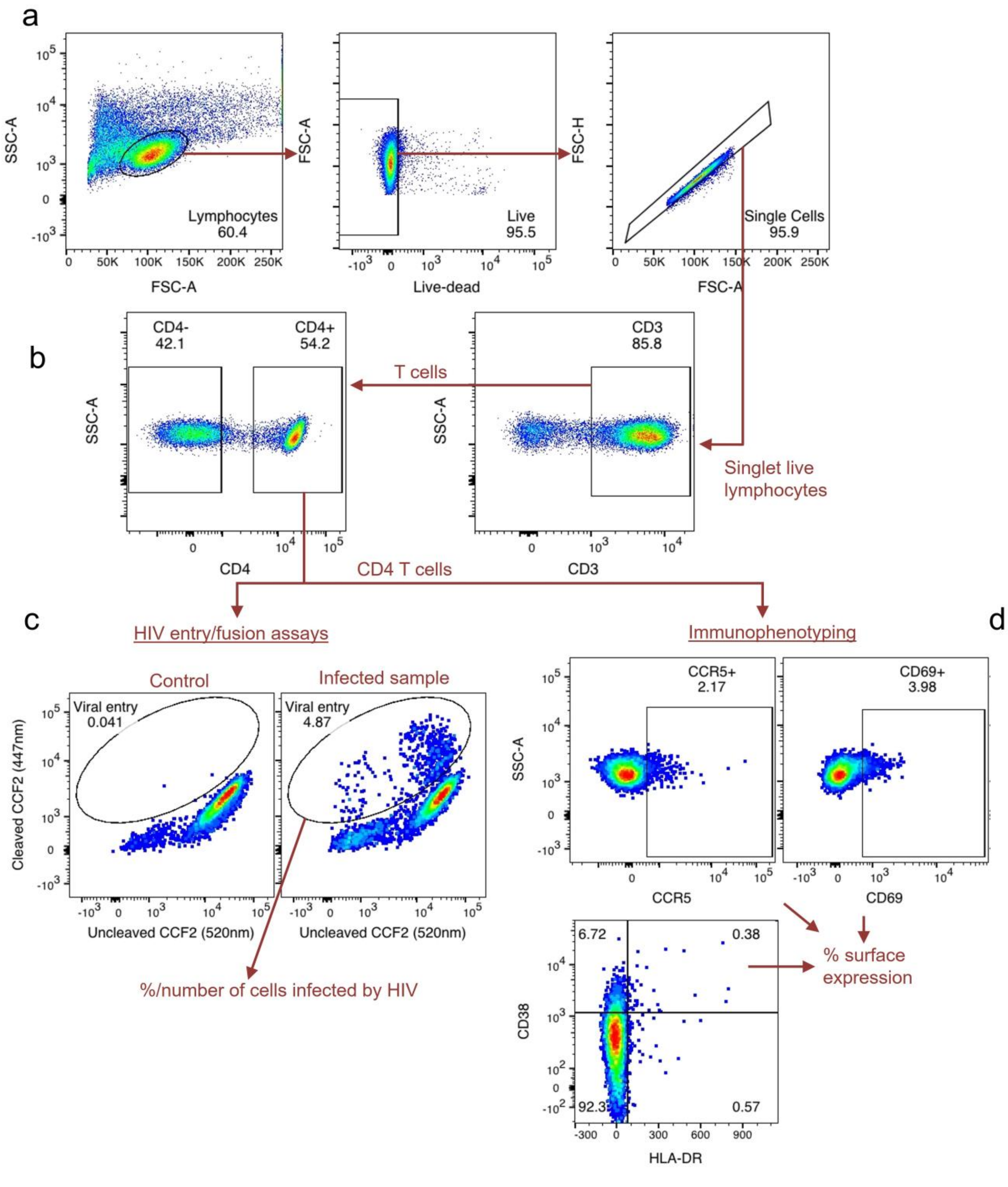

Supplementary Fig. 4. Representative flow cytometry plots and gating strategy for peripheral blood mononuclear cells. a) Gating on single live lymphocytes; b) Gating on CD4 T cells; c) Gating on CD4 T cells infected by HIV pseudovirus; d) Gating on CD4 T cells expressing surface markers CCR5, CD69, CD38/HLA-DR. See **Fig. 2** in main text for a representative $\beta 7^{high}$ plot.

Supplementary Table 8. Changes in HIV entry into cervical and peripheral blood CD4 T cells before (V1) and after schistosomiasis treatment (V2, V3). Source data are provided as a Source Data file.

**Parameter (median)**		**All CCA+ [a]**	***Sm* serology/PCR+ [b]**
% virus entry, cervical CD4 T cells	V1/V2	2.37*	2.37*
	V1/V3	1.59*	1.46*
Number of cervical CD4 T cells with detectable virus	V1/V2	1.21	1.79*
	V1/V3	1.43	1.38
% virus entry, blood CD4 T cells	V1/V2	1.30*	1.31*
	V1/V3	1.23*	1.24*

*Significant change compared to baseline visit, at $p \leq 0.05$, as assessed by paired t-test.

[a] n= 19-22 and 25-31, for paired cervical and blood comparisons, respectively.

[b] n= 12-14 and 17-22, for paired cervical and blood comparisons, respectively.

Median fold changes were calculated first by calculation the corresponding ratio (e.g. V2/V1) for each participant, and then calculating a median of these ratios.

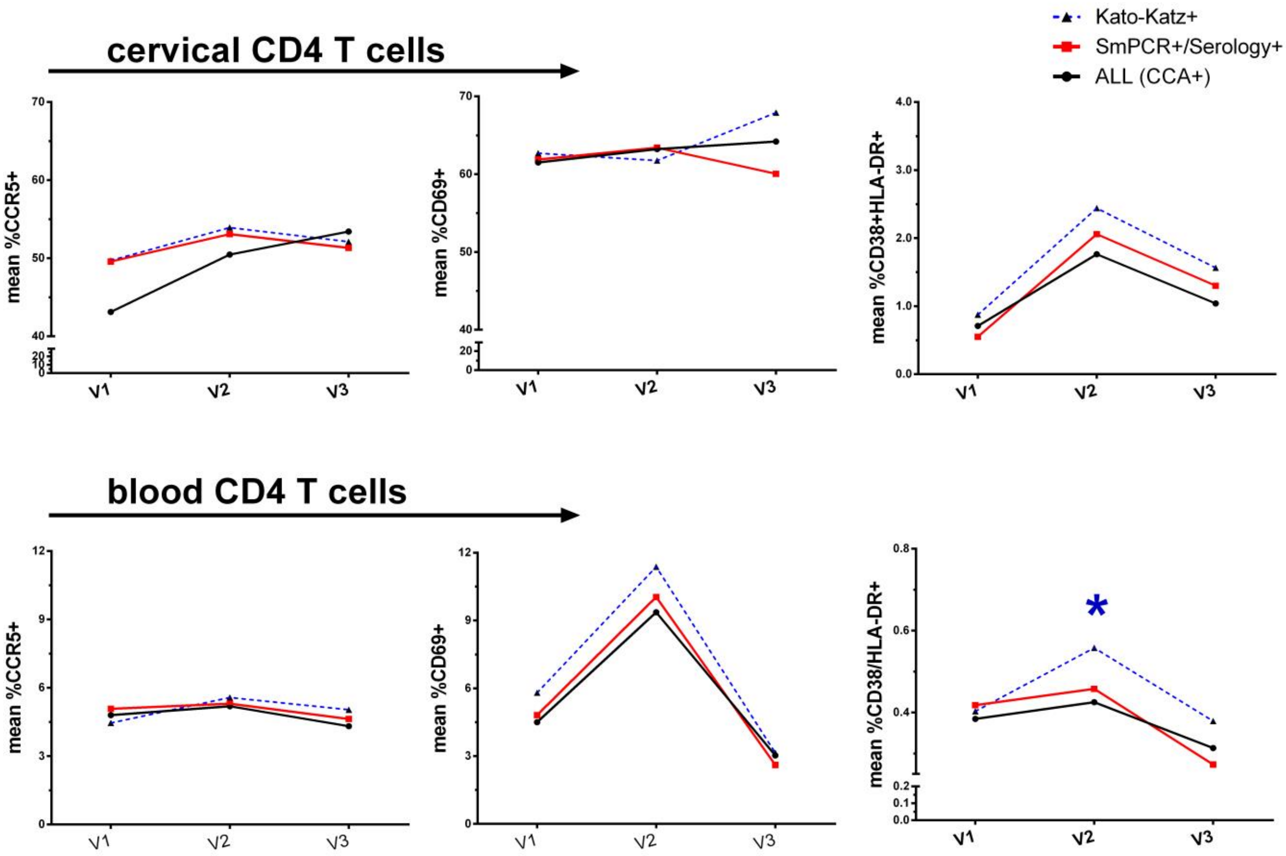

Supplementary Fig. 5. Expression of CCR5 and activation markers CD69 and CD38/HLA-DR on cervical and blood CD4 T cells across study visits. Kato-Katz+ participants tended to exhibit increased levels of CCR5+ and activated CD4 T cells. Blue star denotes a significantly higher (p=0.038, t-test) increase in CD38/HLA-DR expression in Kato-Katz+ participants compared to all CCA+ participants post-treatment. Source data are provided as a Source Data file.

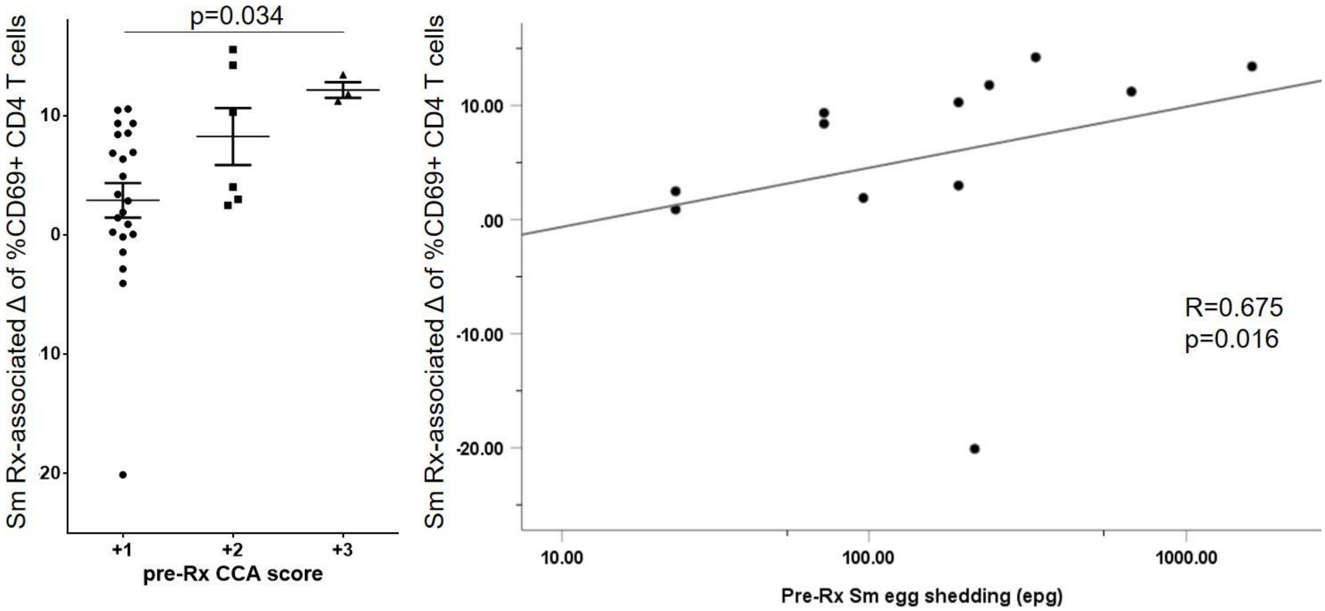

Supplementary Fig. 6. *S. mansoni* treatment-associated elevation of blood CD69+ CD4 T cell levels correlates with pre-treatment infection intensity as assessed by CCA scores (left) and Kato-Katz microscopy (right). Epg=eggs per gram of stool. Significance assessed by one-way ANOVA and Spearman’s rank-order correlation. Source data are provided as a Source Data file.

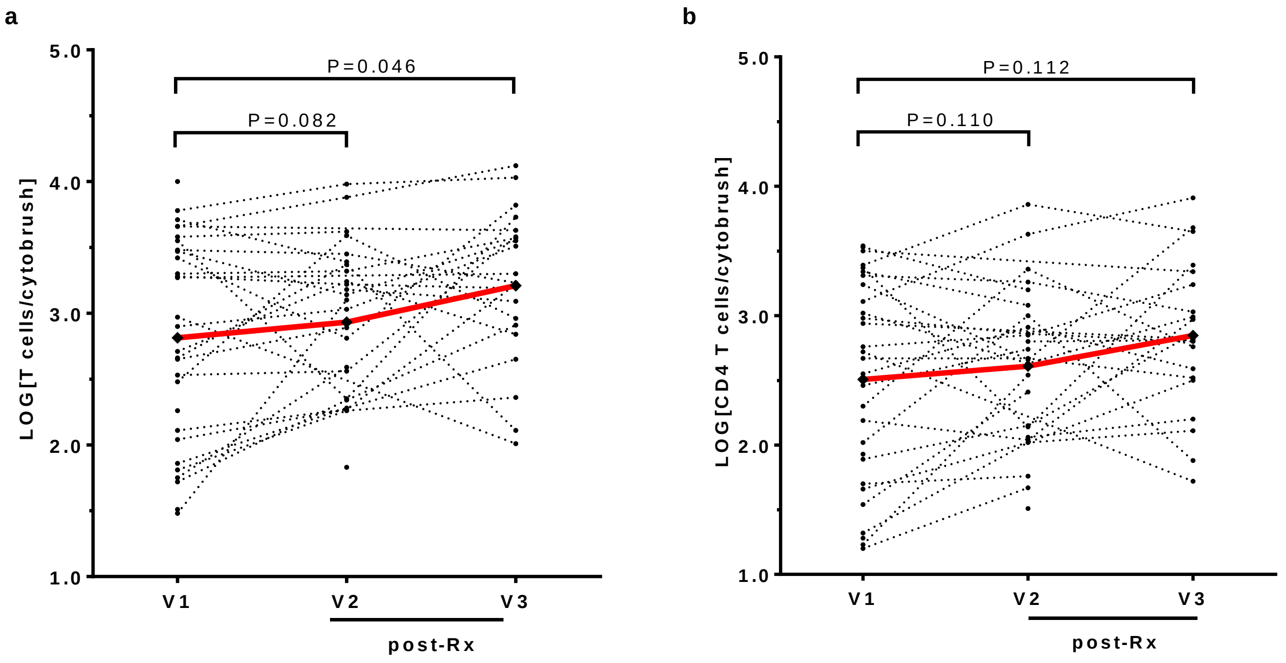

Supplementary Fig. 7. Cervical cytobrush yields across study visits of a) T cells and b) CD4 T cells. Intra-individual difference in cell yields was assessed by paired t-test. Red line depicts means for each study visit. Source data are provided as a Source Data file.

Supplementary Table 9. Cervical cytobrush yields of live T lymphocytes across all study visits (V1-3). Source data are provided as a Source Data file.

		N of live CD3+ cells/cytobrush			N of live CD4+CD3+ cells/cytobrush		
		V1	V2	V3	V1	V2	V3
**Participant Number**		29	26	21	29	26	21
**Mean**		1952	1814	3140	966	985	1494
**Median**		795	1164.5	1686	464	461	663
**Percentiles**	25	119	212	755	63	113	323
	50	795	1164	1686	464	461	663
	75	3305	2370	4028	1898	1046	1956

Supplementary Note 4. Multiplex ELISA on blood plasma and genital secretions

The lower and upper limits of detection and mean concentration values for each cytokine are shown in Supplementary Table 10. Each study participants' samples were run on the same day using the same ELISA plate. When sample's assessed concentration exceeded that of assay ULOD, samples were

diluted to sufficient extent and re-run with corresponding plate controls and paired samples. When sample's assessed concentration was below the assay's LLOD, sample was rerun undiluted.

Supplementary Table 10. Cytokine assay characteristics. LLOD=lower limit of detection, ULOD= upper limit of detection. Mean concentrations are calculated for baseline visit.

Cytokine	Assay LLOD (pg/ml)	Assay ULOD (pg/ml)	Genital secretions (pg/ml): mean (min, max)	Blood (pg/ml): mean (min, max)
V-PLEX MSD Panel				
IL-1α	7.65	350	1295.69 (0, 54633.72)	n/a
IL-1β	0.01	566	383.02 (14.3, 6230.01)	0.09 (0, 4.03)
IL-17	0.246	5,500	15.27 (0, 219.3)	n/a
IL-10	0.03	370	4.78 (0.2, 88.79)	0.65 (0.1, 6.91)
IL-6	0.09	756	126.03 (1.66, 8310.01)	0.49 (0.13, 2.34)
IL-13	0.63	504	45.42 (9.44, 131.35)	0.62 (0.19, 6.99)
IL-4	0.02	249	2.0 (0.32, 14.62)	0.03 (0, 0.08)
IFN-γ	0.12	1,730	23.27 (2.21, 12369.76)	5.68 (2.21, 50.2)
TNF	0.06	373	25.17 (1.69, 1361.95)	1.84 (0.56, 4.27)
IL-12	0.08	496	5.48 (0.6, 46.71)	0.06 (0.02, 0.17)
IL-2	0.11	1,620	14.99 (1.1, 78.83)	n/a
IP-10	0.60	350	165.52 (0.66, 26400)	n/a
Rantes	0.297	2,500	13.63 (0, 248.38)	n/a
MIP-3α	0.835	2,500	69.6 (0, 7618.86)	n/a
MIG	0.52	625	560.35 (0, 13899.84)	n/a
Il-8	0.04	661	n/a	3.09 (0.7, 11.61)
IL-8 (high affinity)	49.5	75,900	15596.06 (159.97, 526404.16)	n/a
MIP-1α	3.94	942	143.97 (16.78, 3989.46)	n/a
MIP-1β	0.001	1,010	83.15 (0, 3702.14)	n/a
U-PLEX MSD Panel				
IFN-α2a	0.301	2,500	3.75 (0, 357.55)	n/a
IFN-β	3.71	100,000	UND*	UND*
Pan IFN alpha Stem Cell ELISA				
Pan IFNα	1.00	316	8.78 (3.86, 32.36)	1.25 (0.21, 8.11)

n/a: not applicable

*Undetectable in >75% of samples

Supplementary Table 11. Baseline characteristics of volunteers who received empiric praziquantel treatment. SmSEA variable is shown as a mean and SEM.

Characteristic	All participants (n=4)
**Sex (N of females)**	3
**Median age (IQR)**	38.5 (33.5-48)
**Urine CCA score**	All negative
**SmSEA serology (OD)***	0.073 (0.025)
**SmPCR**	All negative

*OD: optic density units. Serological optic density (OD) values >0.2 were considered positive.

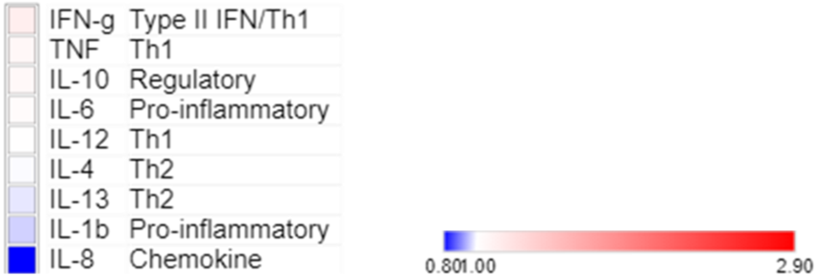

Supplementary Fig. 8. Heatmap of circulating cytokine level changes in schistosomiasis-free volunteers, who received empiric praziquantel treatment (n=4). The heatmap shows ratio of cytokine levels at one month post-therapy compared to pre-treatment baseline. Source data are provided as a Source Data file.

Supplementary Table 12. Pseudovirus entry and CD69 expression data in volunteers who received empiric praziquantel treatment.

Participant #	blood CD4+ T cells			
	Pre-treatment %		One month post-treatment %	
	CD69+	Viral entry	CD69+	Viral entry
1	3.76	0.60	3.98	0.63
2	6.27	0.74	2.92	1.445
3	2.91	1.16	3.73	1.555
4	2.73	1.24	3.25	1.82

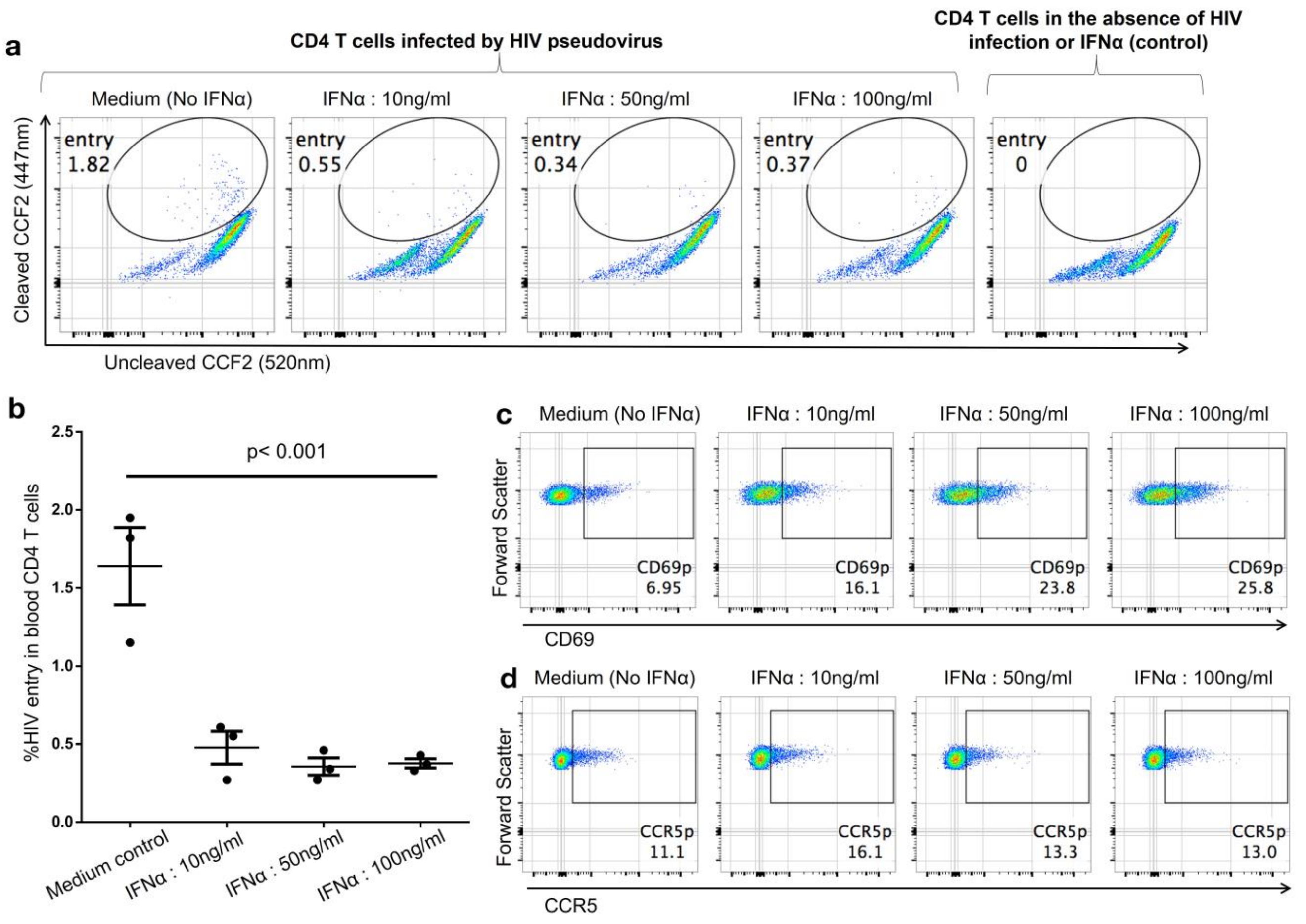

Supplementary Fig. 9. a) Representative flow cytometry plots depicting virus entry into blood CD4 T cells treated with three different doses of IFN-α2a or medium without IFN-α2a (as control). **b)** Changes in virus entry into blood CD4 T cells treated with three different doses of IFN-α2a or medium as control. Dots (n=3 per treatment) represent technical replicates for the same individual. Bars denote standard errors of mean. Significance across treatments assessed by one-way ANOVA ($p<0.05$). **c, d)** Representative flow cytometry plots depicting changes in expression of CD69 (**d**) or CCR5 (**e**) by blood CD4 T cells stimulated by three different doses of IFN-α2a.

Supplementary Note 5. RNA-seq analysis: Effect of *Sm* treatment on the global gene expression

Our RNA-seq exploration of *Sm* treatment-associated changes consisted of a paired comparison of gene expression profiles at one and two months post-treatment versus the baseline (pre-treatment) profile (see Main text methods).

Overall, our DE analysis identified similar proportions of up-/down-regulated genes at V2 (Fig. 4a), but a shift toward down-regulation at V3 (Supplementary Fig. 10a). The immune networks enriched post-treatment included several previously-described mediators of anti-helminth immunity [1, 2], such as the tumor growth factor (TGF)-beta, IL-2, IL-5 and thymic stromal lymphopoietin (TSLP) signaling pathways (Fig. 4b, Supplementary Fig. 10). Consistent with our cytokine findings in blood, IL-10 was down-regulated in PBMC post-treatment. Interestingly, *Sm* treatment was also associated with changes in expression of multiple integrins, including α2, αE and αM ("ITGA2", "ITGAE" and "ITGAM" in Supplementary Fig. 10a) and down-regulation of IL-1 receptor (IL-1R1).

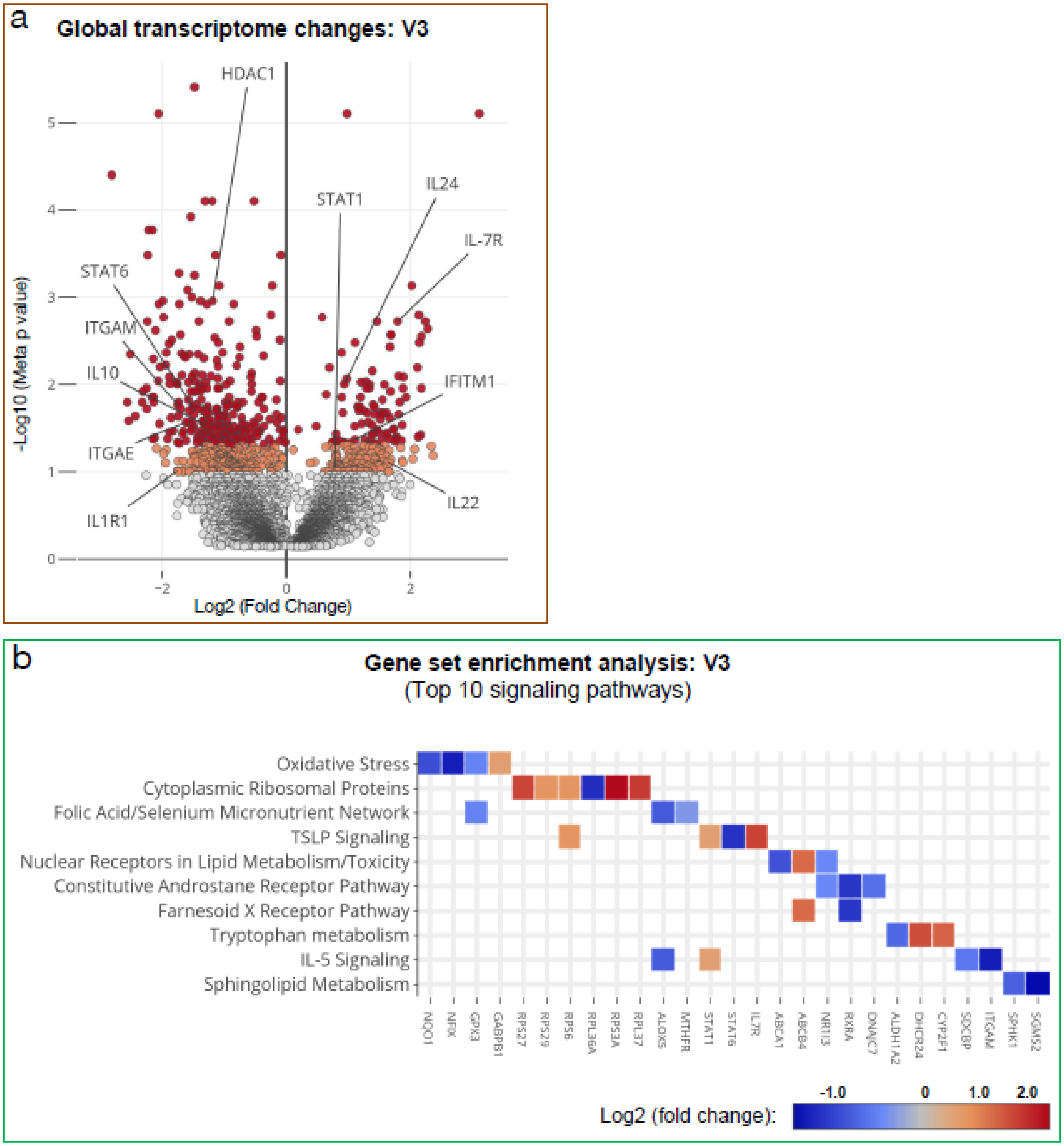

Supplementary Fig. 10. Schistosomiasis treatment-associated PBMC transcriptome changes at 2 months post-schistosomiasis treatment (V3). **a:** Volcano plot depicting the global distribution of log-transformed fold change and meta-analysis p-values for DE gene analysis comparing 2 months post-treatment with baseline. Red dots denote genes with p values ≤0.05; orange dots denote genes with 0.05<p≤0.1. Cumulatively of 687 DE genes (red+orange), 456 (66.4%) were down- and 231 (33. 6%) up-regulated. See the Source Data file for the complete gene list and links to interactive plots. Labels on the volcano plot denote select genes discussed in text; IL1R1 (interleukin 1 receptor 1), ITGA (integrin alpha) -E and -M, IL(interleukin)-10, -22 and -24, STAT (signal transducer and activator of

transcription) 1 and 6, HDAC (Histone deacetylase)-1, IL-7R (interleukin 7 receptor), IFITM (interferon inducible transmembrane protein)-1. **b:** The top 10 enriched pathways identified by the gene set enrichment analysis using all DE genes from the meta-analysis. TSLP: thymic stromal lymphopoietin.

Permutation analysis to test for IRG/IRG-I enrichment

Supplementary Table 13. Frequencies of IFN and IFN-I regulated genes (IRG and IRG-I, respectively) obtained from the Interferome database and used to perform permutation analysis for IRG enrichment

RNAseq dataset	# Total	# IRG	# IRG-I
One-month DEG	1044	296	172
Two-month DEG	688	166	89
Sm-assocated DEG	461	141	73
All genes expressed in the RNAseq dataset	8459	2082	1102

* DEG: differentially expressed genes

The R code used to perform the permutation analysis for IRG and IRG-I enrichment:

```
V2 <- rbinom(1000, 1043, 0.2461)
mean(V2)
hist(V2)
distV2 <- replicate(2000, sample(V2, length(V2), FALSE))
hist(distV2, xlim = c(200,325), col = "blue", breaks = 50)
abline(v = 296, col = "red", lwd = 2)
sum(dist > 296)/2000000  # one-tailed test

V2_T1 <-  rbinom(1000, 1043, 0.1400)
hist(V2_T1)
distV2_T1 <- replicate(2000, sample(V2_T1, length(V2_T1), FALSE))
hist(distV2_T1, xlim = c(100,200), col = "blue", breaks = 30)
abline(v = 172, col = "red", lwd = 2)
sum(distV2_T1 > 172)/2000000  # one-tailed test
```

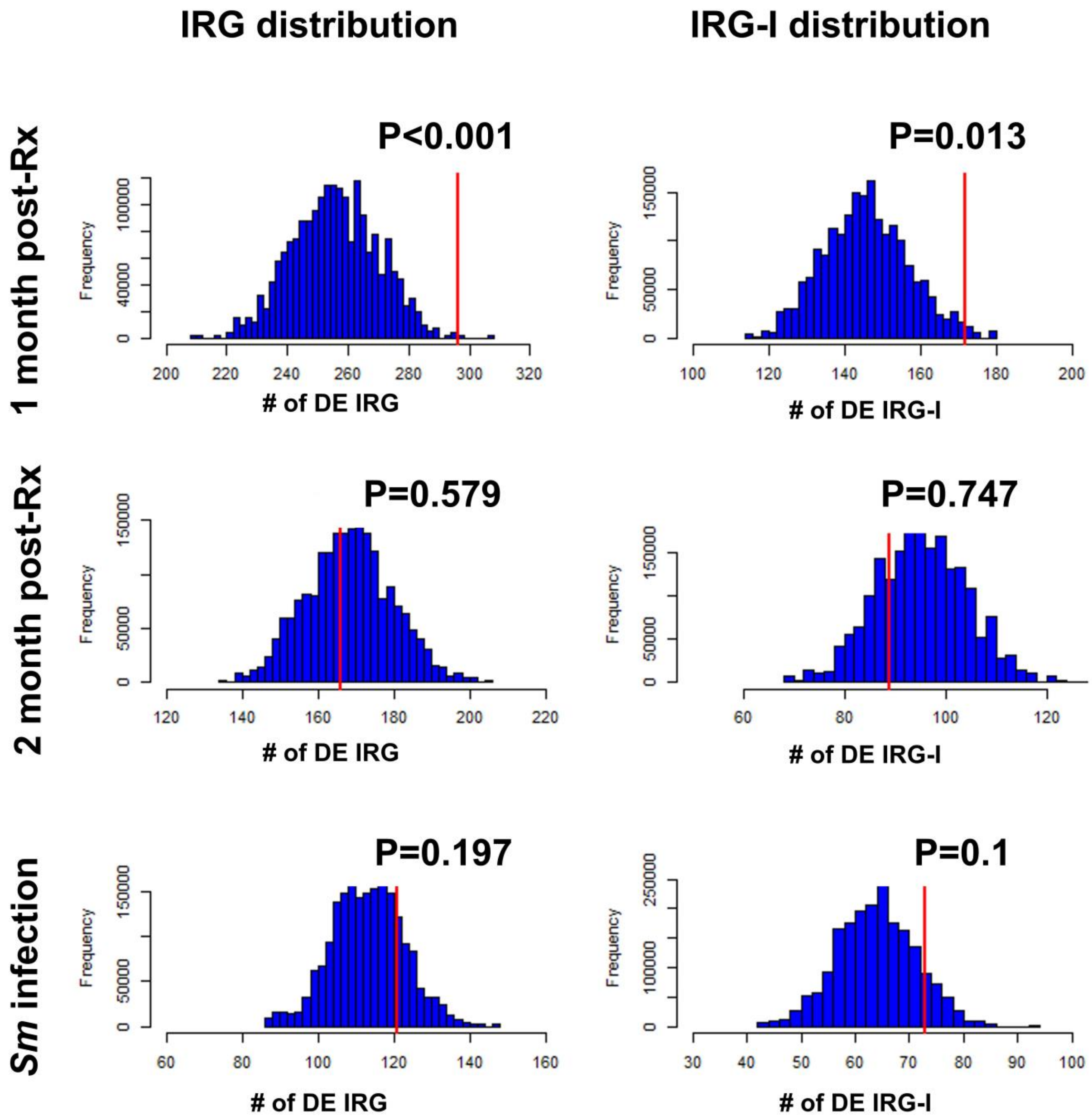

Supplementary Fig. 11. Histograms depicting the permutation-derived null distributions of IFN (not type specific) and IFN-I regulated genes (IRG and IRG-I, respectively) for the differentiall expressed (DE) genes associated with *S.mansoni* treatment (one and two months post-Rx) and with prevalent *Sm* infection. Red vertical lines denote the observed frequency of respective DE genes for each dataset. P values indicate the statistical significance of the observed IRG frequencies compared to the empirically derived gene distribution.

Cell type enrichment using transcriptome data

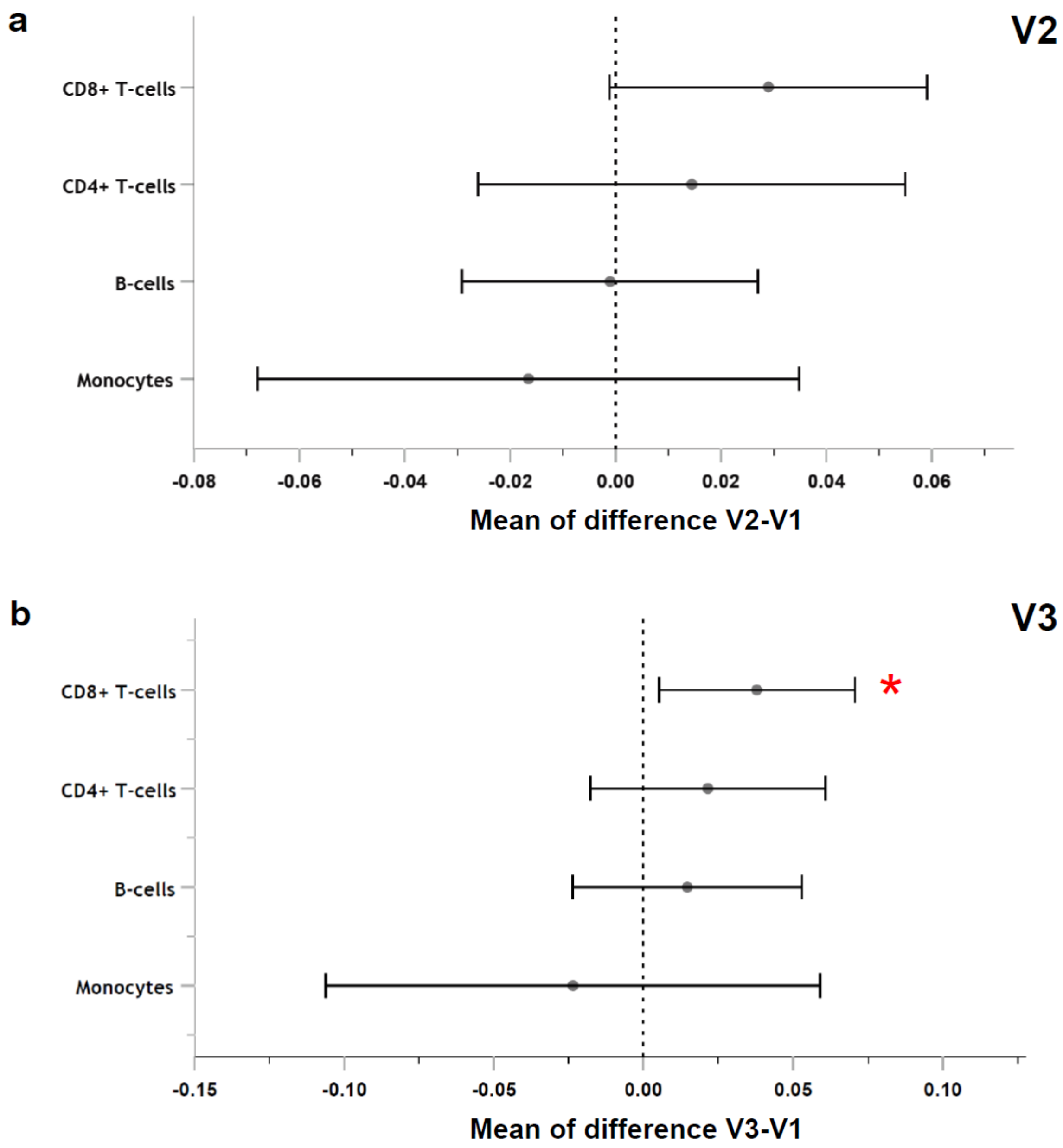

Supplementary Fig. 12. Changes in major lymphocyte subsets and monocytes deduced by Xcell enrichment analysis based on the PBMC RNA-seq counts. T cells, especially CD8 T cells, exhibited most change, whereas B cells remained relatively unchanged post-treatment. Round dots represent mean of difference between study visits and bars represent 95% confidence intervals for the means. Red star denotes a significant difference (p=0.038) as assessed by paired t-test.

Supplementary Note 6. RNA-seq analysis: Effect of *Sm* infection on global gene expression

Our cross-sectional RNA-seq analysis of prevalent *Sm* infection consisted of an unpaired analysis of individuals with and without confirmed *Sm* infection (Main text methods and Supplementary Table 14). Consistent with known suppressive effects of *Sm* on systemic immunity [2], prevalent infection was correlated with a strong signature of protein synthesis down-regulation, altered TNF NF-kB signaling, and differential regulation of pathways associated with cellular proliferation and energy status maintenance (Supplementary Fig. 13b).

Supplementary Table 14. Baseline characteristics of the study participants included in the RNA-seq analysis. Variables are shown as means and SEM.

**Characteristic**	***S. mansoni* + (n=3)**	***S. mansoni* - (n=3)**
**Age**	27.0 (4.36)	32.0 (2.08)
**Urine CCA score**	2.67 (0.33)	0.0 (0.00)
**Kato-Katz (epg)**	728 (190)	0.0 (0.00)
**SmSEA serology (OD)***	1.90 (0.427)	0.052 (0.005)
**SmPCR**	All positive	All negative
**Complete blood counts (cells/ul):**		
**eosinophils**	730.00 (188.77)	123.33 (44.10)
**neutrophils**	196.67 (24.44)	266.67 (55.21)
**basophils**	46.67 (8.82)	50.00 (15.28)
**monocytes**	360.0 (55.08)	336.67 (123.33)
**lymphocytes**	223.33 (95.30)	168.00 (130.77)
**hemoglobin, g/dl**	12.40 (0.513)	13.93 (0.570)

* OD= optic density

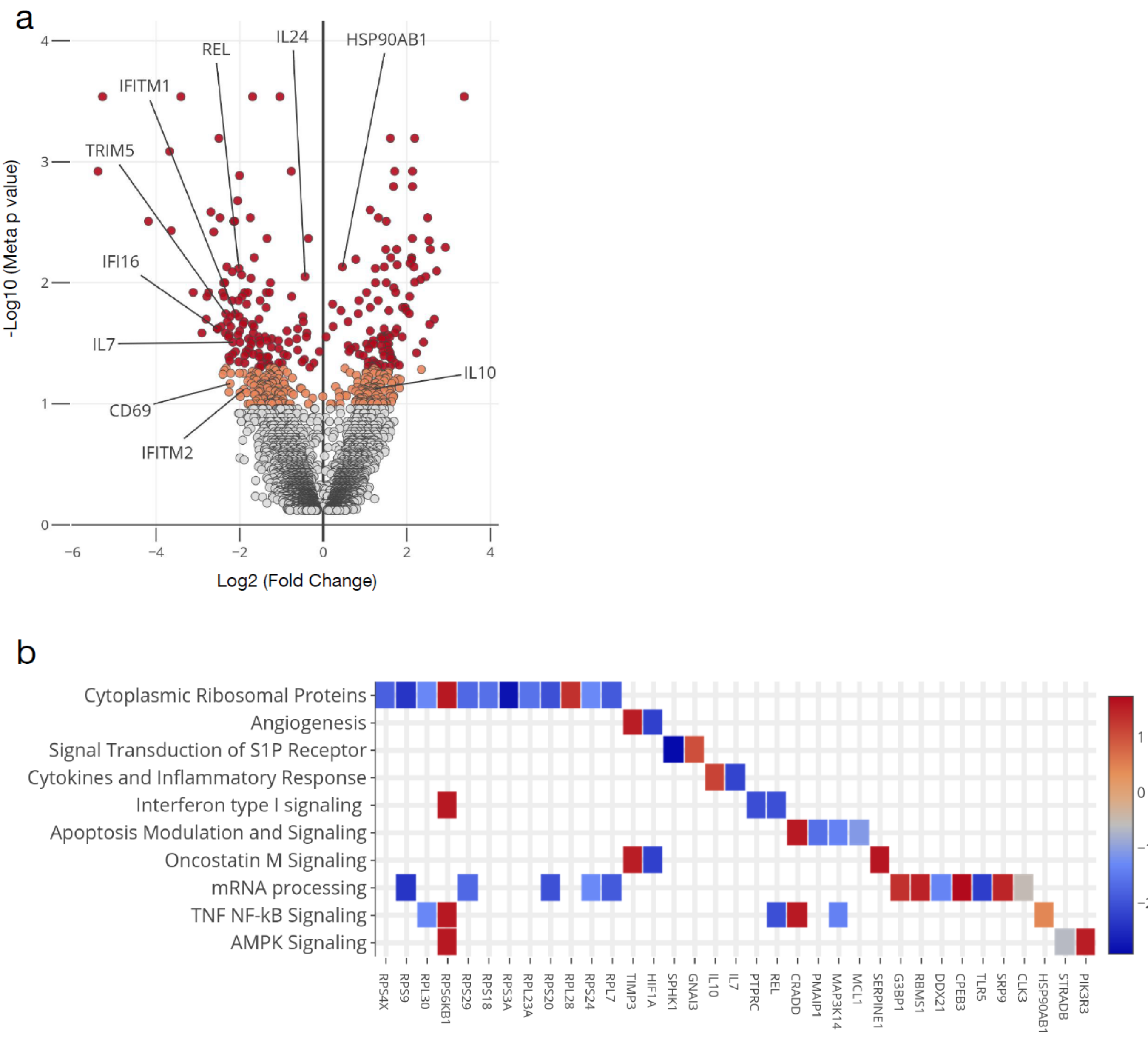

Supplementary Fig. 13. *S. mansoni* infection-associated global PBMC transcriptomic signatures. **a:** Volcano plot depicting the global distribution of log-transformed fold change and meta-analysis p-values for DE gene analysis comparing *Sm*+ versus *Sm*- women. Red dots denote genes with p values ≤0.05; orange dots denote genes with 0.05<p≤0.1. Cumulatively of 460 DE genes (red+orange), 242 (52.6%) were down- and 218 (47.4%) up-regulated. See the Source Data file for the complete gene list and links to interactive plots. Labels on the volcano plot denote select genes discussed in text; IFITM (interferon inducible transmembrane protein)-1 and -2; CD69; interleukin (IL)-7, -10 and 24; gamma-interferon-inducible protein (IFI)-16; tripartite motif-containing protein (TRIM)-5; heat shock protein (HSP)-90AB1. **b:** The top 10 enriched pathways identified by the gene set enrichment analysis using all DE genes from the meta-analysis. S1P: sphingosine 1-phosphate; TNF-kB: Tumour necrosis factor

(TNF)- nuclear factor kappa-light-chain-enhancer of activated B cells (kB); AMPK: AMP-activated protein kinase.

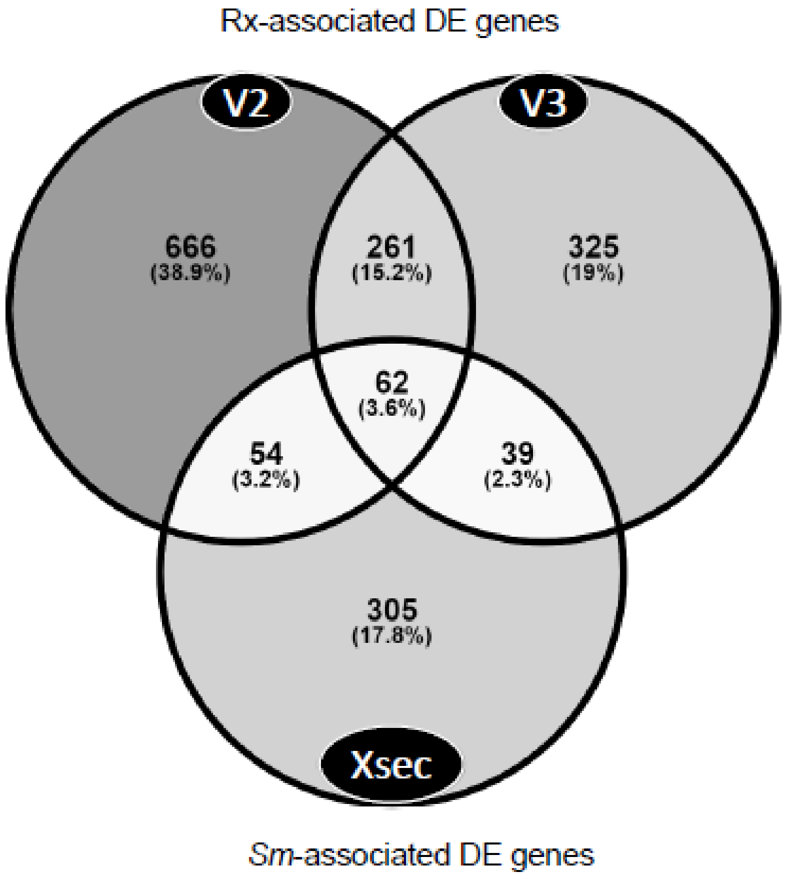

Supplementary Fig. 14. Venn diagram depicting the overlap of DE genes identified in the *S. mansoni* treatment analysis (circles labeled "V2", "V3") and in the cross-sectional *S. mansoni+/S. mansoni-* analysis (circle labeled "Xsec", n=460). There is a total of 155 genes overlapping between the cross-sectional and treatment studies.

Supplementary References

1. Colley DG, Secor WE: Immunology of human schistosomiasis. Parasite Immunol. 2014:36:347-57.
2. McSorley HJ, Maizels RM: Helminth infections and host immune regulation. Clin Microbiol Rev. 2012:25:585-608.
