# Supplementary material for: Schistosoma mansoni treatment reduces HIV entry into cervical CD4+ T cells and induces IFN-I pathways
Source: Nat Commun. 2019 May 24;10:2296. doi: 10.1038/s41467-019-09900-9 (PMC6534541; doi:10.1038/s41467-019-09900-9)
Supplement: Supplementary file 3 — Reporting Summary [file 41467_2019_9900_MOESM3_ESM.pdf]

nature research

Corresponding author(s): Sergey Yegorov

Last updated by author(s): Feb 27, 2019

Reporting Summary

Nature Research wishes to improve the reproducibility of the work that we publish. This form provides structure for consistency and transparency in reporting. For further information on Nature Research policies, see Authors & Referees and the Editorial Policy Checklist.

Statistics

For all statistical analyses, confirm that the following items are present in the figure legend, table legend, main text, or Methods section.

n/a	Confirmed	
☐	☒	The exact sample size (*n*) for each experimental group/condition, given as a discrete number and unit of measurement
☐	☒	A statement on whether measurements were taken from distinct samples or whether the same sample was measured repeatedly
☐	☒	The statistical test(s) used AND whether they are one- or two-sided<br>*Only common tests should be described solely by name; describe more complex techniques in the Methods section.*
☐	☒	A description of all covariates tested
☐	☒	A description of any assumptions or corrections, such as tests of normality and adjustment for multiple comparisons
☐	☒	A full description of the statistical parameters including central tendency (e.g. means) or other basic estimates (e.g. regression coefficient) AND variation (e.g. standard deviation) or associated estimates of uncertainty (e.g. confidence intervals)
☐	☒	For null hypothesis testing, the test statistic (e.g. *F*, *t*, *r*) with confidence intervals, effect sizes, degrees of freedom and *P* value noted<br>*Give P values as exact values whenever suitable.*
☒	☐	For Bayesian analysis, information on the choice of priors and Markov chain Monte Carlo settings
☒	☐	For hierarchical and complex designs, identification of the appropriate level for tests and full reporting of outcomes
☒	☐	Estimates of effect sizes (e.g. Cohen's *d*, Pearson's *r*), indicating how they were calculated

Our web collection on statistics for biologists contains articles on many of the points above.

Software and code

Policy information about availability of computer code

Data collection	Cytokine assay data collection: Discovery Workbench 4.0 (MesoScale Discovery). Flow cytometry assay data collection: FacsDiva (BD)
Data analysis	RNAseq: FASTQC v.0.11.5., HISAT2 v. 2.1.0, SAMTOOLS v. 1.3.1., StringTie v. 1.3.3b, HTSeq v. 0.7.2, RNA-seq 2G, WikiPathways, Enrichr, the Interferome Database, R 3.5.1, xCell webserver. All other statistical analyses: SPSS v. 25, GraphPad Prism v.7.0. Flow cytometry: FlowJo 10.4.1 (TreeStar)

For manuscripts utilizing custom algorithms or software that are central to the research but not yet described in published literature, software must be made available to editors/reviewers. We strongly encourage code deposition in a community repository (e.g. GitHub). See the Nature Research guidelines for submitting code & software for further information.

Data

Policy information about availability of data

All manuscripts must include a data availability statement. This statement should provide the following information, where applicable:

- Accession codes, unique identifiers, or web links for publicly available datasets
- A list of figures that have associated raw data
- A description of any restrictions on data availability

All flow cytometry files were deposited in the FlowRepository database (https://flowrepository.org) under experiment IDs FR-FCM-ZYV5, FR-FCM-ZYVF and FR-FCM-ZYVD. All RNA sequencing files were deposited in the short read sequence archive (http://www.ncbi.nlm.nih.gov/sra) under BioProject ID PRJNA522847. The source data for all figures and tables are included here as a Source Data spreadsheet file. The R code used to perform enrichment analysis is included in the Supplementary Data. The authors declare that all other data supporting the findings of this study are accessible within the article and its Supplementary Information files, or are available from the authors upon request.

Field-specific reporting

Please select the one below that is the best fit for your research. If you are not sure, read the appropriate sections before making your selection.

☒ Life sciences ☐ Behavioural & social sciences ☐ Ecological, evolutionary & environmental sciences

For a reference copy of the document with all sections, see nature.com/documents/nr-reporting-summary-flat.pdf

Life sciences study design

All studies must disclose on these points even when the disclosure is negative.

Sample size	Power calculation was performed based on the standard deviation of the difference in viral entry (primary endpoint of the clinical trial) in repeated measures obtained within an individual. Based on these preliminary calculations, recruitment of n=35 participants would have allowed us to detect a 24% difference in viral entry at β=0.8.
Data exclusions	No data were excluded from the analysis
Replication	Experimental replication was performed where possible and all attempts at replication were successful.
Randomization	Randomization was not performed, since the clinical trial assessed changes before and after antischistosomal therapy within the same group of study participants.
Blinding	Blinding was not performed since all study participants belonged to one experimental group.

Reporting for specific materials, systems and methods

We require information from authors about some types of materials, experimental systems and methods used in many studies. Here, indicate whether each material, system or method listed is relevant to your study. If you are not sure if a list item applies to your research, read the appropriate section before selecting a response.

Materials & experimental systems

n/a	Involved in the study	
☐	☒	Antibodies
☐	☒	Eukaryotic cell lines
☒	☐	Palaeontology
☒	☐	Animals and other organisms
☐	☒	Human research participants
☐	☒	Clinical data

Methods

n/a	Involved in the study	
☒	☐	ChIP-seq
☐	☒	Flow cytometry
☒	☐	MRI-based neuroimaging

Antibodies

Antibodies used	CCR7 (CD197) BV605, clone G043H7, Biolegend, #353224<br>CD4 BV650, clone SK3, BD #563875<br>CD38 BV711, clone HIT2, Biolegend, #303528<br>CD3 BV785, clone OKT3, Biolegend, #317330<br>HLA-DR AF700, clone L243, Biolegend, #307626<br>CCR5 (CD195) PE CF594, clone 2D7, BD #562456<br>Integrin B7 PEcy5, clone FIB504, BD #551059<br>CD69 PE cy7, clone FN50, Biolegend, #310912<br>Other reagents:<br>Live Dead Far Red, Thermo Fisher/Invitrogen, #L10120
Validation	All of these antibodies are commercially available and validated for flow cytometry applications. Dilutions used for each assay are based on the vendor's suggested concentrations and titrations performed in our laboratory.

Eukaryotic cell lines

Policy information about cell lines

Cell line source(s)	HEK293T17 cells were obtained from ATCC (Cat. number CRL-11268).
Authentication	Cells were authenticalted by ATCC

Mycoplasma contamination	Cell lines were not tested for mycoplasma
Commonly misidentified lines (See ICLAC register)	The original cell line was obtained from ATCC. This cell line is optimal for retrovirus production and was only used to produce pseudovirus stocks.

Human research participants

Policy information about studies involving human research participants

Population characteristics	This study involved the use of endocervical and blood samples from HIV-negative women (aged 18-45) diagnosed with schistosomiasis. Blood samples were also collected from schistosomiasis-uninfected donors.
Recruitment	Participants with a clearly positive urine circulating cathodic antigen (CCA) test result (scored as "+1" or above) were invited to participate in the study and then screened for inclusion/exclusion criteria. Exclusion criteria were HIV infection, malaria infection, current pregnancy, genital ulceration, active menstruation, positive for classical STIs (Neisseria gonorrhoeae, Chlamydia trachomatis, Treponema pallidum or Trichomonas vaginalis), or deemed by study staff to be unlikely to comply with study requirements.
Ethics oversight	All study procedures were approved by the Uganda Virus Research Institute Research and Ethics Committee, the Uganda National Council for Science and Technology, and the Institutional Review Board at the University of Toronto. Written informed consent was obtained from all participants.

Note that full information on the approval of the study protocol must also be provided in the manuscript.

Clinical data

Policy information about clinical studies

All manuscripts should comply with the ICMJE guidelines for publication of clinical research and a completed CONSORT checklist must be included with all submissions.

Clinical trial registration	#NCT02878564
Study protocol	https://clinicaltrials.gov/ct2/show/NCT02878564
Data collection	The trial was conducted in Entebbe, Uganda in March-October 2016. The initial screening of participants occurred at the Uganda Virus Research Institute (UVRI) community outposts offering free HIV testing and counselling to the general population of Lake Victoria communities. Consenting HIV-uninfected Ugandan women aged 18-45 years from these communities were tested for schistosomiasis by urine CCA. All CCA+ participants were scored by two technologists using a published scoring scheme and those scored as "+1" or above were invited to participate in the study and then screened for inclusion/exclusion criteria (see section above). Genital samples were collected at the UVRI-IAVI clinic along with venous blood in the following order: cervico-vaginal secretions, vaginal swabs, and two endocervical cytobrushes. Sample processing and flow cytometry data acquisition were performed at the UVRI-IAVI laboratory.
Outcomes	The primary endpoint of the clinical trial was the change in the percentage and number of endocervical CD4+ T cells susceptible to HIV pseudovirus entry after treatment of schistosomiasis. The secondary outcomes included assessment of changes in: i) the percentage of blood CD4+ T cells susceptible to HIV pseudovirus entry after treatment of schistosomiasis, ii) the phenotype of endocervical and blood CD4+ T cells after treatment of schistosomiasis, iii) genital proinflammatory cytokine levels after treatment of schistosomiasis.

Flow Cytometry

Plots

Confirm that:

☒ The axis labels state the marker and fluorochrome used (e.g. CD4-FITC).

☒ The axis scales are clearly visible. Include numbers along axes only for bottom left plot of group (a 'group' is an analysis of identical markers).

☒ All plots are contour plots with outliers or pseudocolor plots.

☒ A numerical value for number of cells or percentage (with statistics) is provided.

Methodology

Sample preparation	Mononuclear cells were extracted from cervical cytobrushes and blood. Endocervical cytobrush was inserted into the cervical os, rotated through 360̊, and stored in R10 medium at 4°C until processing. Cells from two cytobrushes were eluted, combined, passed through a 100-µm filter, washed, and divided into two equal aliquots for use in the flow cytometry and virus entry assays at the UVRI-IAVI laboratory. Blood was collected by venipuncture into ACD (16ml) and EDTA (4ml) vacutainers (BD). Peripheral blood mononuclear cells (PBMC) were isolated from ACD blood by layering onto Ficoll Histopaque (Sigma) and centrifuging at 400g for 30 min followed by reconstituting at 10 million cells/ml in Roswell Park Memorial Institute (RPMI) 1640 medium (Sigma) with 10% heat-inactivated fetal bovine serum (FBS) (Wisent Inc., Canada). Approximately two million PBMC were used in flow cytometry assays.

Instrument	Flow cytometry was performed on either BD LSR-II or BD LSR Fortessa X-20 (BD Biosciences) cytometers at the UVRI-IAVI laboratory or University of Toronto, respectively.
Software	FACSDIVA V8.0.1 (BD) was used for data acquisition, FlowJo (TreeStar Inc.) was used for data analysis
Cell population abundance	Cell sorting was not performed in this study.
Gating strategy	The gating strategy is provided in the Supplementary Figs 3-4. First, FSC-A vs. SSC-A plots were used to gate on lymphocytes. Then, dead cells were excluded using live-dead dye vs. FSC-A gating. Next, single cells were defined by FSC-H vs FSC-A gating. Subsequently, T cells were defined by gating on CD3+ live singlet cell populations (CD3 vs. SSC-A). T cells were then sub-gated into CD4- and CD4+ populations (CD4 vs. SSC-A). Gating for CCR5, CD38/HLA-DR, CD69 and integrin b7 was performed on CD4+ T cells using FMO controls. Pseudovirus entry gating was performed on CD4+ T cells using an uninfected sample as FMO control.

☒ Tick this box to confirm that a figure exemplifying the gating strategy is provided in the Supplementary Information.
